# Support for 5G Mission-Critical Applications in Software-Defined IEEE 802.11 Networks

**DOI:** 10.3390/s21030693

**Published:** 2021-01-20

**Authors:** Pedro H. Isolani, Daniel J. Kulenkamp, Johann M. Marquez-Barja, Lisandro Z. Granville, Steven Latré, Violet R. Syrotiuk

**Affiliations:** 1 Department of Computer Science, University of Antwerp–imec, 2000 Antwerp, Belgium; steven.latre@uantwerpen.be; 2 School of Computing, Informatics, and Decision Systems Engineering, Arizona State University, Tempe, AZ 85281, USA; dkulenka@asu.edu (D.J.K.); syrotiuk@asu.edu (V.R.S.); 3 Department of Electronics–ICT, University of Antwerp–imec, 2000 Antwerp, Belgium; johann.marquez-barja@uantwerpen.be; 4 Institute of Informatics–INF, Federal University of Rio Grande do Sul, Porto Alegre, Rio Grande do Sul 91501-970, Brazil; granville@inf.ufrgs.br

**Keywords:** SDN, MAC management, airtime-based network slicing, traffic shaping, user association, IEEE 802.11 networks, SD-RAN, MCDA

## Abstract

With the emergence of 5G networks and the stringent Quality of Service (QoS) requirements of Mission-Critical Applications (MCAs), co-existing networks are expected to deliver higher-speed connections, enhanced reliability, and lower latency. IEEE 802.11 networks, which co-exist with 5G, continue to be the access choice for indoor networks. However, traditional IEEE 802.11 networks lack sufficient reliability and they have non-deterministic latency. To dynamically control resources in IEEE 802.11 networks, in this paper we propose a delay-aware approach for Medium Access Control (MAC) management via airtime-based network slicing and traffic shaping, as well as user association while using Multi-Criteria Decision Analysis (MCDA). To fulfill the QoS requirements, we use Software-Defined Networking (SDN) for airtime-based network slicing and seamless handovers at the Software-Defined Radio Access Network (SD-RAN), while traffic shaping is done at the Stations (STAs). In addition to throughput, channel utilization, and signal strength, our approach monitors the queueing delay at the Access Points (APs) and uses it for centralized network management. We evaluate our approach in a testbed composed of APs controlled by SD-RAN and SDN controllers, with STAs under different workload combinations. Our results show that, in addition to load balancing flows across APs, our approach avoids the ping-pong effect while enhancing the QoS delivery at runtime. Under varying traffic demands, our approach maintains the queueing delay requirements of 5 ms for most of the experiment run, hence drawing closer to MCA requirements.

## 1. Introduction

5G networks are designed with the goal of fulfilling the expectations of more stringent QoS support [1]. MCAs, such as autonomous cars, industrial automation, and smart cities, require high-speed connections, enhanced reliability, and low latency. A typical use case scenario that requires Ultra-Reliable Low Latency Communication (URLLC) is the process automation and remote control for reactive flows in a digital factory [2]. Although the date rate of such use cases is relatively low, the End-to-End (E2E) latency is expected to remain under 50 ms. In addition to using 5G Radio Access Technology (RAT), MCAs may also exploit the opportunity to offload traffic into IEEE 802.11 networks [3,4,5,6], which makes dealing with stringent QoS requirements in IEEE 802.11 networks of the utmost importance. Traditional network management solutions and techniques cannot deal with such dynamic environments and their requirements well. The introduction of SDN enabled new levels of innovation and automation that also appear to be appropriate for IEEE 802.11 management [7].

Network slicing is advocated as an appropriate abstraction for network virtualization and flexible resource provisioning [8,9,10]. As expected, the SDN paradigm has also been applied in wireless networks. In fact, the 3GPP 5G architecture is embracing the Control/User Plane Split (CUPS) (a cornerstone of SDN) as one of the fundamental enablers for network programmability and E2E network slicing [1]. As a result, SDN-tailored systems are envisioned to ease the creation of logical and isolated wireless networks via slice abstractions. In IEEE 802.11 networks, network slicing allows for the isolation of network resources and traffic among users and services [11]. To this end, portions of airtime are used for slices in order to achieve a more precise Resource Allocation (RA) mechanism. SDN allows for network slices to be dynamically instantiated, modified, and terminated, facilitating network innovation and simplifying network management [12].

Despite the wide range of research efforts addressing network slicing and SDN to enhance resource utilization [4,13,14,15,16,17,18,19,20], deciding how to efficiently allocate, control, and manage both users and slices remains challenging. In this paper, we tackle three outstanding problems. First, research efforts in network slicing have addressed airtime fairness, traffic isolation, and throughput guarantees; for the support of MCAs, our approach also addresses latency-related metrics. Secondly, network slicing has focused on Downlink (DL) transmissions; MCAs may also generate Uplink (UL) traffic, so our approach jointly assesses both. Third, proposals for user re-association often focus on minimizing the average number of STAs assigned to an AP and maximizing the overall throughput of the network and Received Signal Strength Indicators (RSSIs); because of the latency constraints of MCAs, we include queueing delay in our re-association decision.

In previous work, we evaluated the impact of runtime slice reconfiguration on the E2E latency and we exploited the potential of slice orchestration to guarantee latency-related requirements [18,19]. We developed and integrated queueing delay measurements into the formulation of a QoS optimization problem, and we proposed a delay-aware approach for performing runtime MAC management via airtime-based network slicing and user association using MCDA in the IEEE 802.11 SD-RAN [21]. There, we provided a solution that is capable of load balancing flows and enhancing QoS delivery at the Radio Access Network (RAN). However, only DL flows were considered. In this paper, we extend our delay-aware approach to consider both DL and UL flows. In this context, UL and DL flows are characterized as the traffic generated from STAs to APs and from APs to STAs, accordingly. When dealing with both DL and UL flows, slice management and user association are also impacted.

In summary, the contributions of our paper include:

1. we extend the network control to the IEEE 802.11 end-devices with a programmable agent, which is capable of performing monitoring and traffic shaping, and we propose a traffic shaping algorithm that controls them via a centralized controller;
2. we extend our network slicing and user association algorithms to consider satisfying the QoS of flows in both UL and DL directions; and,
3. we conduct a performance analysis of our approach comparing it to a state-of-the-art user association algorithm [5]. We evaluate both of the approaches in a real-world testbed with three APs, controlled by an SD-RAN and a backhaul SDN controller, and six STAs served by QoS and Best-Effort (BE) flows in both UL and DL directions. Our results show an improved load balancing of flows across APs and QoS guarantees via centralized RAN slicing and traffic shaping at the end-devices.

The remainder of this paper is organized, as follows. In Section 2, we review related work. This is followed, in Section 3, by an overview of our system. In Section 4, we describe our approach, providing the algorithms for user association, network slicing, and traffic shaping. Section 5 presents our testbed, workload, and results of our experimentation. Finally, in Section 6, we summarize and describe future work.

## 2. Related Work

Ensuring QoS in wireless networks is a longstanding research challenge that has become more complex with the advent of 5G [22]. To cope with the stringent requirements of MCAs, industry and standardization bodies have been fostering research towards reliable and improved QoS delivery. In IEEE 802.11 networks, QoS has been pursued based on the adaptation of Enhanced Distributed Channel Access (EDCA) parameters, traffic shaping, and slice scheduling. On the other hand, user association algorithms have been developed while using the SDN paradigm to enhance load balancing, mobility support, and fairness. In this section, we review the major efforts in these areas.

### 2.1. Resource Allocation and QoS Support

Table 1 presents an overview of the related work in IEEE 802.11 networks, summarizing how each approach targets network management, QoS support, the RA method, and the tools used for experimentation. After the IEEE 802.11e amendment [23] established the foundations for traffic prioritization, many investigations focused on queuing management as a means to enhance QoS [24,25,26,27]. Most work concentrated on scheduling schemes incorporating the length of the traffic queues, the time to serve a packet, or the time waiting on the scheduler. Later, with the improvements in radio resource utilization provided by the IEEE 802.11n amendment [28], researchers focused on channel optimization and fairness (e.g., modifying or predicting the Aggregated MAC Service Data Unit (A-MSDU) behavior) [29,30,31,32,33]. However, such proposals required modifications to the driver (e.g., frame formats) and are no longer compliant with the standard.

Several research efforts involved infrastructure sharing via network slicing [13]. Apart from experimentation isolation and analysis [38,42], most of the approaches consisted of airtime-based RA mechanisms for IEEE 802.11 network virtualization [14,35,36]. The focus of airtime scheduling has been extensively studied as a means to overcome the well-known IEEE 802.11 Performance Anomaly [43]. Without slicing capabilities, all STAs would share the available radio resources equally only if they experience similar channel conditions. Otherwise, when STAs are located far away from the APs and use a lower bit rate, the result is a performance degradation that is perceived by all STAs.

Nakauchi et al. [35] and Guo et al. [36] presented similar proposals. Their focus was on an airtime RA method through the control of EDCA parameters, such as the Contention Window (CW) size and the Transmission Opportunities (TXOPs). Although proper traffic isolation was not achieved, the authors have worked to schedule DL packets based on throughput requirements. Other authors [39,41] presented alternative solutions to the problem, performing traffic shaping to limit the resource usage of each slice and by the use of a queuing model with feedback control. However, proper slice isolation was not guaranteed, due to changes in performance of one STA affecting others.

Other research efforts [34,37] concentrated on EDCA parameter adaptation to guarantee the airtime of slices. However, there was only control over the UL traffic. Moreover, both of the approaches were only evaluated in simulation and did not provide support for RA or traffic isolation, which is crucial in achieving and ensuring high data rates. Recently, several proposals [4,14,15,16,17,18,19,20] addressed network slicing in IEEE 802.11 networks. Richart et al. [14] proposed a mechanism that assigns airtime portions to each slice as a resource to be shared. Later, Richart et al. [20] presented an enhanced version of such scheduling with capacity limits, which is capable of achieving precise queueing delay for slices on an AP. However, that work was only assessed in simulation. On the other hand, there were also several practical implementationsm [4,15,16,17], but runtime slice orchestration based on latency requirements was not addressed.

Coronado et al. [4] proposed a framework that enables programmable and dynamic E2E network slicing over heterogeneous RANs. The framework was deployed on a real-world testbed, showing that slices can be dynamically defined and STA/slice traffic isolation can be achieved. In previous work [18,19], we evaluated the impact of runtime slice reconfiguration on the E2E latency and exploited the potential of slice orchestration to guarantee latency-related requirements. We developed and integrated queueing delay measurements into the formulation of a QoS optimization problem and proposed a delay-aware approach for performing runtime network slicing and MAC management using MCDA in IEEE 802.11 SD-RANs [21]. There, we provided a solution capable of load balancing flows and enhancing QoS delivery at The RAN. However, only DL flows were considered. In this paper, we extend our delay-aware approach to consider both DL and UL flows.

### 2.2. User Association and Load Balancing

Extensive research has been conducted on user association and load balancing in IEEE 802.11 networks. Although there are several distributed approaches, most recent efforts concentrated on centralized network management solutions [3,5,44,45,46,47,48,49,50,51,52,53,54,55]. The SDN paradigm allows for researchers to introduce new mechanisms without having to modify the IEEE 802.11 standard. In addition, SDN brings significant improvements, particularly in terms of QoS awareness. Consequently, several proposals have benefited from the centralized view of SDN to provide more sophisticated and intelligent solutions. For instance, instead of STAs simply associating with the AP with highest RSSI, other metrics can be considered. In addition, researchers have used the Light Virtual Access Point (LVAP) abstraction, in which a physical AP uses different LVAPs for communication with each STA and this, in turn, avoided problems that are caused by legacy handover algorithms, such as unnecessary re-associations and connection disruptions.

Because we focus on centralized handover management and load balancing among devices using the same network technology, we summarize the related work in this specific research domain. Table 2 presents the most recent efforts on centralized-horizontal handover management and load balancing solutions that were proposed for IEEE 802.11 networks. Murty et al. [44] enabled proactive handovers that were based on RSSIs gathered from both STAs and AP. Targeting location-awareness, they developed a system with several Application Programming Interfaces (APIs) that allow such RSSIs to be available at a centralized controller. Similarly, Murty et al. [45] extended such APIs to support a wider range of input measurements. Similar work can be found in [46].

Apart from work conducted via simulations [47,52], testbed experimentation has also been carried out [50,55]. By considering the content requested by the STAs and their throughput requirements, authors have assessed user association and multicast delivery jointly. Consequently, the authors presented significant performance enhancements over the default STA-driven approaches. The AP selection problem has been addressed in both proactive [54,55] and reactive [49,53] manners. In [54], enhanced mobility support and throughput enhancements were targeted through a supervised learning model with a wider range of input parameters, including the predicted location of STAs, RSSIs, and load of the APs. Moreover, to ensure the preservation of the QoS, the negative impact of STA re-association, i.e., the handover cost, was considered. In this way, STAs avoided handovers between AP with similar loads or experiencing similar channel conditions. This minimized ping-pong effects, while achieving higher overall throughput as compared to the IEEE 802.11 standard handover algorithm.

Coronado et al. [3] addressed the user association and load balancing problem with a joint channel selection and user association scheme. With a constraint programming algorithm, possible collision domains among APs were isolated. As also stated in recent research [51,55], when considering AP load and channel conditions is decisive to avoid network performance degradation. As a result, a user association scheme is proposed in order to detect when traffic is not efficiently distributed and perform handovers to the STAs causing performance issues. In order to perform user-association, the algorithm first computed the channel utilization for each AP and the average channel occupancy across all the APs of the network. Subsequently, if there is a significant difference between the channel utilization and any occupancy ratio, the algorithm selected candidate APs to handover that have the lowest result of the product between current occupancy ratio and the RSSIs of its neighboring APs.

Based on the work presented in [3], Gómez et al. [5] proposed a user association algorithm for enhanced resource allocation. They listed a few limitations of the previous work, including: static channel assignment, excessive number of handovers under low load levels, and the fact that the user re-association process considered neither the average RSSIs of STAs nor the deterioration of signal quality. Therefore, an adjustment of The AP load threshold is proposed, which reduces The excessive number of handovers under low load levels, and they introduced two new indicators that triggered the re-association of STAs. The user association algorithm is based on three indicators: average RSSI of an AP, AP load, and channel occupancy. The first indicator referred to the average of the UL RSSIs for all STAs connected to the APs. The second represented the load of the APs in the network, while the third represented the load of the channels, in which APs are operating. Despite these metrics, the user association algorithm checked the indicators sequentially, and decisions are based on only one indicator at a time.

According to Coronado et al. [3], the majority of user association mechanisms target (*i*) the minimization of the number of STAs per AP, the maximization of the average signal quality, or (*ii*) the maximization of the average throughput of the network. We identify that little attention is given to latency-restricted services, and latency-related metrics are not considered within the handover decision-making processes. Today’s MCAs are latency-sensitive, motivating the need for considering the delay metrics. In this paper, in addition to other metrics, we monitor the queueing delay at the APs as part of our centralized network management. In this manner, we enhance SD-RAN resource utilization and QoS delivered to support of MCAs.

## 3. System Overview

**Figure 1:**
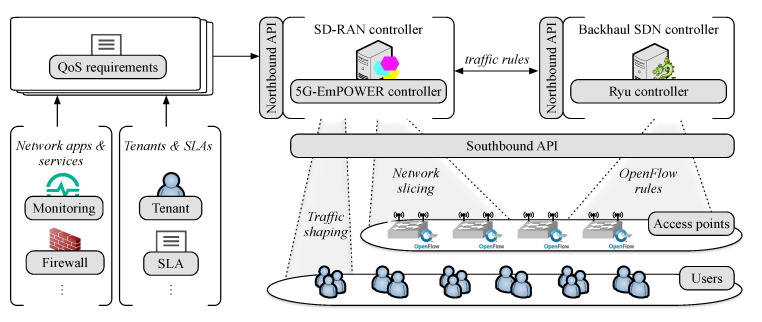
SDN-enabled layered network architecture.

Figure 1 illustrates our SDN-enabled layered network architecture. Multiple *tenants*, i.e., virtual operators or service providers, share the infrastructure and they have their specific Service Level Agreement (SLA). These SLAs are translated into QoS requirements, in which the network has to support, e.g., minimum throughput, maximum allowed E2E latency, and acceptable packet loss ratio.

The IEEE 802.11 RAN consists of a set of APs responsible for delivering data from different services to/from several users (STAs) in the network. Each AP has resources to be shared and, therefore, managed. To control resources utilization and ensure QoS delivery at the RAN, we propose the use of network slicing. We focus on QoS within a slice as being a service, i.e., Quality of Service Slicing (QoSS), as defined by Richart et. al. [14]. To perform network network-triggered handovers, airtime-based network slicing, and traffic shaping, our approach relies on the 5G-EmPOWER platform (https://github.com/5g-empower/5g-empower.github.io), which includes the 5G-EmPOWER SD-RAN controller, a backhaul implementation of the SDN controller Ryu, and a programmable agent that runs at each AP. The IEEE 802.11 interface of the AP is set monitor mode for radio measurements collection. We extended the SD-RAN controller to allow flow demands and QoS requirements to be informed. Thus, the SD-RAN controller can calculate the expected throughput and verify the QoS. The network intelligence is implemented at the SD-RAN controller, which communicates with the APs at the data plane through its southbound interface using a persistent Transmission Control Protocol (TCP) connection. This communication is given by the *OpenEmpower protocol* that, besides monitoring, allows for operations, such as the reassignment of the available resources among slices.

The programmable agent at the APs, in turn, consist of two components: An OpenvSwitch (https://www.openvswitch.org/) instance that operates under the supervision of the OpenFlow-enabled SDN controller and a Click modular router [56] instance implementing the IEEE 802.11 data-path with a *hypervisor*. The hypervisor sits on top of the standard Linux IEEE 802.11 stack of the APs, therefore, acts as a software overlay. In this manner, the SD-RAN controller can request the backhaul controller to tag traffic matching a certain flow through the definition of traffic rules (i.e., OpenFlow rules [57]). Therefore, slices are mapped by the hypervisor according to the Service Set Identifier (SSID) and the Differentiated Services Code Point (DSCP). The SSID is the name of a IEEE 802.11 network and the DSCP determines the priority of each IP packet. Multiple flows can be mapped into a single slice/ the hypervisor is in charge of creating, monitoring, and managing network slices according to traffic rules, ensuring performance isolation and efficient resource utilization.

**Figure 2:**
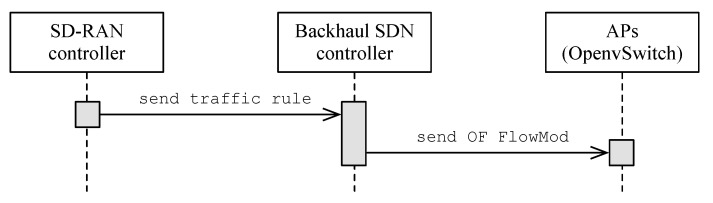
Sequence diagram of the traffic rule creation process.

**Figure 3:**
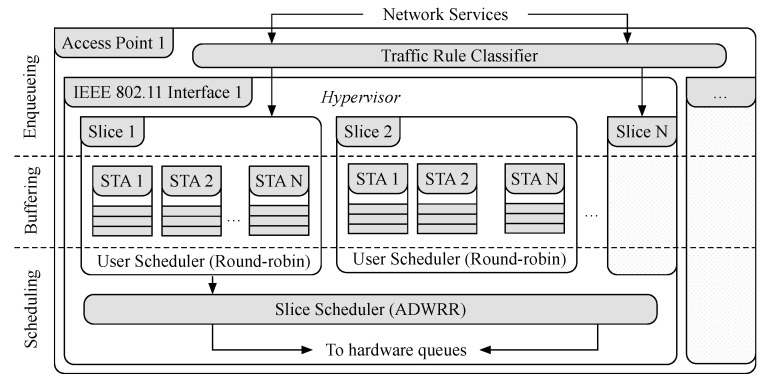
Simplified slice queue structure and data traffic flow in an Access Point (AP).

Figure 2 illustrates the sequence of communication from the SD-RAN controller to the OpenvSwitch instance at the APs. First, the SD-RAN controller sends a message to the backhaul SDN controller containing the traffic rule description. The backhaul SDN controller then takes the traffic rule description and installs the defined traffic rules in each of the APs as OpenFlow rules. At each AP, the IEEE 802.11 interface defines the minimum chunk of wireless resources an STA can use, including the network interface identifier (e.g., MAC address), operating channel (e.g., 1, 6, 11), and the type of channel (e.g., High Throughput (HT) 20 MHz, Very High Throughput (VHT) 40 MHz). Figure 3 shows a simplified queue structure along with the data traffic flow within an AP.

First, frames from slices are classified into queues based on the definition of the traffic rules (e.g., OpenFlow rules [57]).Each traffic rule contains multiple aggregation buffers, one for each user in the slice. These aggregation buffers are scheduled while using the Round Robin policy and subsequently are dequeued following the Airtime Deficit Weighted Round Robin (ADWRR) scheduling algorithm [4]. In the rest of this section, we present the ADWRR scheduling algorithm in Section 3.1. Subsequently, in Section 3.2, we present our proposed frame tracking functionality to monitor the queueing delay of slices. Finally, in Section 3.3, we present the implementation details of the programmable agent in order to perform dynamic traffic shaping at the STA.

### 3.1. ADWRR Scheduling Algorithm

The ADWRR scheduling algorithm is responsible for assigning a fraction of the airtime to each traffic rule according to its relative priority. The main purpose of ADWRR is to consider the cost of transmitting a frame with regard to the resources that have to be allocated to it [4]. In this case, the cost of transmitting a frame depends on its length and the actual channel conditions that are experienced by its receiver. The hypervisor only serves traffic rules whose expected transmission time, as estimated by a rate control algorithm (e.g., Minstrel [58]), is smaller than a deficit counter. The scheduler only iterates upon active queues/slices; inactive queues/slices do not cause any performance degradation to the system. With ADWRR, the quantum of an individual slice can be adjusted independently, which allows for the airtime of the slice to be controlled dynamically. In this way, a larger quantum can be assigned to a slice supporting services with stricter performance requirements, allocating it more radio resources.

### 3.2. Monitoring Queueing Delay at APs

**Figure 4:**
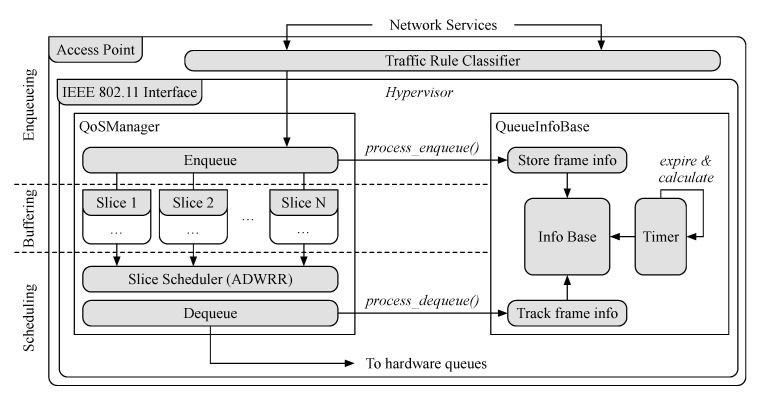
Queueing delay monitoring in the simplified queue structure and data flow in an AP.

The hypervisor is implemented while using the click modular router. Click is a framework for writing multi-purpose packet processing engines [56] and it is used to implement the wireless STA/AP frame exchange. Figure 4 illustrates the main elements involved in the computation of the queueing delay of slices along with a simplified version of the hypervisor implementation.

We introduced a custom click element *QueueInfoBase* that keeps track of the frames dequeued at the AP. This element maintains the frame information, including the slice identifier and the timestamps when a frame is enqueued and dequeued. Average queueing delay is calculated by the element periodically according to a configurable timer, according to which frames were dequeued during the period. We set this period to be one second in our system, and outdated frame information is removed. To make use of the new statistics, we extended both the OpenEmpower protocol (used for the controller and AP communication) and the SD-RAN controller. Several handler apps at the controller periodically request and calculate the needed metrics, which are maintained at the controller, and a Simple Moving Average (SMA) and Simple Moving Median (SMM) of the last ten measurements are calculated. The queueing delay statistics are utilized by the management algorithms we implement for our user association, traffic shaping, and network slicing.

### 3.3. Shaping and Monitoring UL Traffic with a Programmable Agent

To allow for the UL traffic, generated by the STAs, to be shaped by the centralized SD-RAN controller, we introduce a programmable agent at the STAs. Traffic shaping is widely used in order to improve latency and bandwidth of flows by delaying others [59]. The agent receives commands from the network controller and applies traffic shaping on all of the traffic leaving the IEEE 802.11 wireless interface of the STAs.

**Figure 5:**
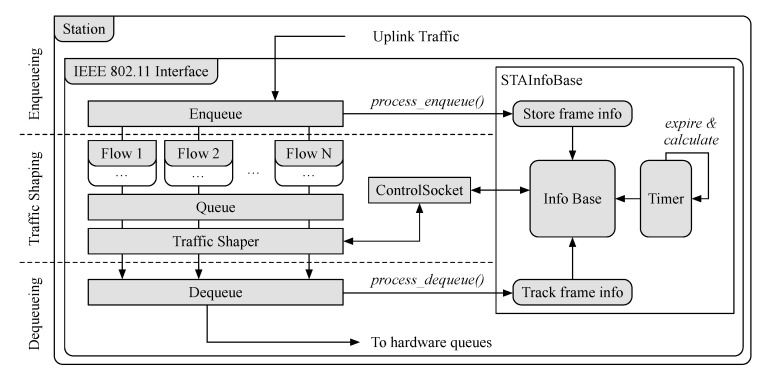
Traffic shaping in the simplified queue structure and data flow in an Station (STA).

Figure 5 illustrates the main elements that make up the agent implementation at the STAs. This agent is also implemented while using the click modular router and it behaves as a software overlay on top of the IEEE 802.11 data-path. In addition to performing traffic shaping, our implementation keeps track of the enqueued and dequeued frames at the STAs. Specifically, just before frames are enqueued, the element Enqueue sends the timestamp when the frame was enqueued along with the frame identifier to the element STAInfoBase. This element, in turn, stores the identifier and timestamp, and then compares it to when the frame is dequeued to obtain the delay. If frames are not dequeued (i.e., they are dropped by the shaper), a packet loss counter is updated. As with the agent on the APs, the delay and loss statistics are updated every second, and outdated information is removed.

The SD-RAN controller communicates with the agents on STAs through a persistent TCP connection, while using the ControlSocket element. This element enables external access to other element handlers, allowing for read and write operations to be performed. Such operations can be performed on the STAInfoBase element, for example, enabling the controller to read statistics regarding the configuration of the traffic shapers. Moreover, the controller can also adjust the shaping for an STA that is negatively impacting other QoS flows in the network. We define a minimum and maximum value for the shaper, to keep traffic within a range of values. Our minimum value, 1 Mbps, was chosen in order to ensure we do not block an STA from transmitting, and our maximum value, 100 Mbps, was chosen to be well above the capacity of the channel.

## 4. Delay-Aware Sdn-Based Approach

In this section, we present our delay-aware approach for network slicing and MAC management while using MCDA in IEEE 802.11 SD-RANs. MCDA is a sub-discipline of operations research that evaluates multiple conflicting criteria in decision making and finds the best alternative from a set of available alternatives. We apply MCDA whenever we want to decide to which AP an STA is assigned, according to the high-level objectives of balancing the AP load while considering the delay constraints of any QoS MCAs. In order to enhance the QoS delivery, we complement our approach by performing network slicing at the IEEE 802.11 SD-RAN and traffic shaping at the end-devices.

Next, we formulate our criteria for MCDA, and present our user association, slicing, and shaping algorithms. All of the notations introduced are listed in Table 3 for convenience.

### 4.1. Load Balancing Problem Formulation Using MCDA

The IEEE 802.11 RAN consists of a set *B* of APs, being responsible for delivering services to a set *T* of STAs. Within an AP, *n* services have to be delivered, hence, *n* slices are instantiated. Each service is instantiated in a slice, with $S^{b}$ denoting the slices of AP b∈B. Therefore, each STA *t* is served by a subset of the slices of $S^{b}$. In addition, each slice *s* has a quantum $Q^{s}$ that defines the amount of airtime added to the deficit counter of the slice scheduler. Services are characterized by bidirectional data flows; therefore, we also consider flows coming from the STAs, i.e., the UL flows. Each flow *f* from the STAs is measured at a given AP and, therefore, considered in our load balancing problem, with $F^{t}$ denoting the flows of STA *t*. The traffic shaping value $\lambda^{t}$ is configured at STA *t*, $\lambda_{\mathrm{LOSS}}^{t}$ is the loss in frames/sec that is introduced by this shaper, and $D^{t}$ is its queueing delay.

We select six criteria for MCDA to evaluate for an AP *b*: (*i*) the overall channel load $\theta^{b}$ in B/s; (*ii*) the total measured throughput $\mu^{b}$ of both UL flows and DL slices; (*iii*) the total expected throughput $\mu_{\mathrm{EXP}}^{b}$ of both UL flows and DL slices; (*iv*) the total measured queueing delay $D^{b}$; (*v*) $t_{\mathrm{RSSI}}^{b}$, the RSSI perceived at *b* from STAs within range; and, (*vi*) an indicator variable $t^{b}$, which evaluates to true if STA *t* is associated with AP *b*. The first four criteria are minimized in order to avoid resource overuse. We use (*iv*) to avoid APs with a high number of active or overflowing queues. This reduces the chance of a Network Interface Card (NIC) overload and channel saturation. The last two criteria are maximized to improve the chances of using higher data rates, and of fewer connection disruptions, respectively.

The overall measured throughput, $\mu^{b}$, comprises both UL and DL load measured at *b*. The dequeueing rate (i.e., throughput) of slice *s* is denoted $\mu^{s}$, while $\mu^{f,t}$ denotes the measured throughput of flow *f* measured from STA *t*. Therefore,

(1) $$\mu^{b}=\sum_{s\in S^{b}}\mu^{s}+\sum_{t\in T}\sum_{f\in F^{t}}\mu^{f,t}\;\cdot \;t^{b},\;\forall b\in B.$$

On the other hand, the overall expected throughput $\mu_{\mathrm{EXP}}^{b}$ of *b* is:

(2) $$\mu_{\mathrm{EXP}}^{b}=\sum_{s\in S^{b}}\sum_{t\in T}\mu_{\mathrm{EXP}}^{s,t}\;\cdot \;t^{b}+\sum_{t\in T}\sum_{f\in F^{t}}\mu_{\mathrm{EXP}}^{f,t}\;\cdot \;t^{b},\;\forall b\in B,$$

where $\mu_{\mathrm{EXP}}^{s,t}$ is the expected throughput for STA *t* in slice *s* and $\mu_{\mathrm{EXP}}^{f,t}$ is the expected UL throughput for flow *f* from STA *t*. Note that the expected throughput is calculated based on flow demands, while the actual throughput is measured at the APs. Furthermore, the overall queueing delay $D^{b}$ of *b* is calculated as the aggregated queueing delay of its slices. Thus,

(3) $$D^{b}=\sum_{s\in S^{b}}D^{s},\;\forall b\in B,$$

where $D^{s}$ is the average queueing delay within slice *s*. Some slices, in turn, specify QoS requirements for throughput and queuing delay of certain flows, denoted $\mu_{\mathrm{QoS}}^{s}$ and $D_{\mathrm{QoS}}^{s}$, accordingly. In addition to DL traffic, STAs might also require QoS support. Therefore, $\mu_{\mathrm{QoS}}^{f,t}$ specify the throughput requirements of a flow *f* from STA *t*. Last but not least, $t^{b}$ is a binary variable, indicating whether the STA *t* is associated with AP *b*. Therefore, for all b∈B and all t∈T:

(4) $$t^{b}=\left\{\begin{array}{cc} 1 & \mathrm{if}\;\mathrm{STA}\;t\;\mathrm{is}\;\mathrm{associated}\;\mathrm{with}\;\mathrm{AP}\;b,\\ 0 & \mathrm{otherwise}. \end{array}\right.$$

The weight of each criterion depends on the flow type, either QoS or BE. We use the Analytic Hierarchy Process (AHP) [60] to inform our selection of weights for each flow type, and then tune the resulting weights to avoid the ping-pong effect. In order to avoid STAs performing handovers between APs under similar channel conditions and resource utilization, we use the $t^{b}$ criterion. Because we maximize this criterion in our MCDA formulation, a higher preference is given to the candidate AP to which STAs are already connected. In this manner, when handover decisions involve APs in such conditions, STAs tend to remain connected to their current APs, thus avoiding the ping-pong effect. Another consideration is that we want BE flows to be more likely to undergo handovers than QoS ones, because handovers are detrimental to meeting the QoS requirements. Therefore, we provide different weights for QoSs flows in order to account for this need. Table 4 lists the MCDA criteria and the resulting weights by flow type ($W_{\mathrm{BE}}$ and $W_{\mathrm{QoS}}$).

Several guidelines exist for choosing the appropriate method to solve an MCDA problem [61]. Given that our problem has quantitative weights, a quantitative scale of comparisons, no uncertainty, and it is characterized by a complete ranking, we select the Technique for Order of Preference by Similarity to Ideal Solution (TOPSIS) method [62]. TOPSIS ranks the alternative solutions by minimizing the distance to the positive ideal solution and maximizing the geometric distance from the negative ideal solution. Next, we show how to use the solution to our MCDA problem in order to perform load balancing among the APs.

### 4.2. Using MCDA in the User Association Algorithm

Our user association algorithm is given in Algorithm 1. At a high level, this algorithm periodically decides to which AP each STA should be assigned while using MCDA. In each round, TOPSIS ranks the candidate APs for STAs to perform handovers and the best AP is selected. Thereafter, the algorithm triggers the handovers. We now describe the algorithm in more detail.

In order to avoid handover decisions being made for STAs in the same order each round, the algorithm randomizes the order of STAs in each reconfiguration loop (line 11). Additionally, handovers are only considered for STAs with active flows. Subsequently, the expected throughput of *t* is calculated based on its active DL slices and UL flows, followed by the selection of MCDA weights (lines 13 and 14). The weights $W^{t}$ vary by flow type: The $W_{\mathrm{QoS}}$ defines the weights used if an STA *t* is being served by a flows in a QoS slice or have UL flows with QoS requirements; otherwise, $W_{\mathrm{BE}}$ is used. In addition to the statistics, the algorithm calculates the expected throughput of each AP according to STAs distribution and their flow demands (line 16).

Using expected throughput can cause the ping-pong effect, because the expected throughput of the AP to which an STA is connected will be higher than when the STA is not connected. To avoid this problem, we consider the expected throughput of the AP without the load of the STA under consideration. Therefore, we subtract the expected throughput of an STA *t* from the overall expected throughput of the AP with which it is connected (line 18). This prevents the expected throughput of *t* from affecting its own handover decisions. TOPSIS is then used to solve the MCDA problem; it returns $b_{\mathrm{BEST}}$, the highest-ranked AP according to the criteria (line 20). An STA only undergoes a handover if it is not associated with its top-ranked AP $b_{\mathrm{BEST}}$ (line 22).

First, the algorithm gathers the statistics monitored from all APs (line 9). For each AP, this includes the overall channel load, the measured throughput as the sum of the throughput of all slices and all flows measured from the STAs associated with it, the measured queueing delay of all slices, and the measured RSSI. For brevity, we use $b_{\mathrm{STATS}}$ to represent all of these statistics of an AP *b*.

Given that APs are usually set to operate on different channels, our algorithm avoids excessive handovers per reconfiguration loop, so as to minimize the impact of switching channels on throughput and delay of flows. In case of a handover between APs operating on different channels, the Channel Switch Announcement (CSA) mechanism is triggered. CSA is defined by the IEEE 802.11h amendment in order to enable APs to announce switching to a new channel before their transmission begins on that channel. Beacon messages containing the CSA information are sent to The STA before it switches to the new channel. This allows STAs, which support CSA, to move to the new channel with minimal downtime.

Our algorithm also avoids executing multiple handovers on a single AP in each reconfiguration loop. Except for the expected throughput, the measured statistics do not reflect the network re-configurations instantaneously and this might cause inappropriate handover decisions. Therefore, in each reconfiguration loop, $B_{\mathrm{HANDOVER}}$ is the set of APs in which handovers have been performed. Hence, handovers only happen for STAs, in which their current AP or highest-ranked AP did not undergo handovers in the present reconfiguration loop (line 21).
**Algorithm 1** User Association Algorithm**Input:**
 1: every                     ▹ configuration loop interval (20 s used)
 2: $C,W_{\mathrm{QoS}},W_{\mathrm{BE}}$                  ▹ set of MCDA criteria and weights
 3: $\forall s\in S^{b}:D_{\mathrm{QoS}}^{s}$                ▹ max queueing delay of each slice *s*
 4: $\forall s\in S^{b}:\mu_{\mathrm{QoS}}^{s}$                   ▹ min throughput of each slice *s*
 5: $\forall f\in F,\forall t\in T:\mu_{\mathrm{QoS}}^{f,t}$         ▹ min-avg throughput of each flow *f* from STA *t*
 6: $\forall s\in S^{b}:\mu_{\mathrm{EXP}}^{s,t}$                ▹ expected throughput of each STA *t*


 7: **loop** every
 8:     **for each**
b∈B
**do**                     ▹ iterate over all APs
 9:         $b_{\mathrm{STATS}}\leftarrow$ getRBStats*(b)*

 10:     $B_{\mathrm{HANDOVER}}\leftarrow \emptyset$
 11:     **for each**
t∈
random.shuffle(*t*)
**do**               ▹ iterate over all STAs
 12:         **if**
getActiveFlows(*t*) **then**
 13:            $\mu_{\mathrm{EXP}}^{t}\leftarrow$
getSTAExpectedLoad(*t*)
 14:           $W^{t}\leftarrow$
getSTAWeights(*t*)
 15:           **for each**
b∈B
**do**                    ▹ iterate over all APs
 16:                $\mu_{\mathrm{EXP}}^{b}=$
getRBExpectedLoad$(b,\mu_{\mathrm{EXP}}^{t})$
 17:                **if**
$t^{b}=true$
**then**
 18:                $\mu_{\mathrm{EXP}}^{b}=\mu_{\mathrm{EXP}}^{b}-\mu_{\mathrm{EXP}}^{t}$
 19:                ${\mathrm{TOPSIS}.}_{\mathrm{ALTERNATIVE}}(C,W^{t},\mu_{\mathrm{EXP}}^{b},b_{\mathrm{STATS}})$
 20:            $b_{\mathrm{BEST}}\leftarrow {\mathrm{TOPSIS}.}_{\mathrm{BESTALTERNATIVE}}\left(\right)$
 21:            **if**
$t^{b_{\mathrm{BEST}}}\neq true$
**and**
$\left\{b,b_{\mathrm{BEST}}\right\}\;\notin B_{\mathrm{HANDOVER}}$
**then**
 22:                
doHandover
$\left(b_{\mathrm{BEST}}\right)$                ▹ handover to AP $b_{\mathrm{BEST}}$
 23:                $B_{\mathrm{HANDOVER}}\overset{+}{\leftarrow }\left\{b,b_{\mathrm{BEST}}\right\}$


 24:
 25: **function** 
getSTAWeights(*t*)
 26:     **for each**
$f\in F^{t}$
**do**        ▹ iterate over all flows generated at a given STA
 27:         **if**
$\mu_{\mathrm{QoS}}^{f,t}$
**then return**
$W_{\mathrm{QoS}}$
 28:     **for each**
$s\in S^{b}$
**do**                ▹ iterate over all slices of an AP
 29:         **if**
$D_{\mathrm{QoS}}^{s}$**or**$\mu_{\mathrm{QoS}}^{s}$
**then return**
$W_{\mathrm{QoS}}$
 30:     **return**
$W_{\mathrm{BE}}$
 31: 
 32: **function** 
getSTAExpectedLoad(*t*)
 33:     $\mu_{\mathrm{EXP}}^{t}\leftarrow 0$
 34:     **for each**
$s\in S^{b}$
**do**              ▹ iterate over all slices of an AP
 35:         $\mu_{\mathrm{EXP}}^{t}=\mu_{\mathrm{EXP}}^{t}+\mu_{\mathrm{EXP}}^{s,t}$
 36:     **for each**
$f\in F^{t}$
**do**       ▹ iterate over all flows generated at a given STA
 37:         $\mu_{\mathrm{EXP}}^{t}=\mu_{\mathrm{EXP}}^{t}+\mu_{\mathrm{EXP}}^{f,t}$
 38:     **return**
$\mu_{\mathrm{EXP}}^{t}$
 39:
 40: **function** getRBExpectedLoad($b,\mu_{\mathrm{EXP}}^{t}$)
 41:     $\mu_{\mathrm{EXP}}^{b}\leftarrow 0$
 42:     **for each**
t∈T
**do**                    ▹ iterate over all STAs
 43:         **if**
$t^{b}=true$
**then**
 44:            $\mu_{\mathrm{EXP}}^{b}=\mu_{\mathrm{EXP}}^{b}+\mu_{\mathrm{EXP}}^{t}$

 45:     **return**
$\mu_{\mathrm{EXP}}^{b}$


### 4.3. Network Slicing Algorithm

Algorithm 2 is used for adapting the network slice configurations at runtime. In addition to the quantum adjustments, this adaptation is based on three thresholds: the maximum queueing delay of the QoS slices, the minimum throughput of the QoS slices, and the minimum throughput of the QoS flows that were measured from the STAs. The network slicing algorithm aims to satisfy the QoS requirements of the QoS flows in both DL and UL directions. By reallocating resources from the BE slices, the algorithm delays the traffic dequeued from the BE slices in favor of the QoS-constrained traffic. Periodically, the algorithm checks, for each AP, whether the requirements of all QoS slices and all QoS UL flows measured from STAs that are associated with it are met. When all of the requirements are met, the quantum value of the BE slices, sharing the AP, is increased by a factor of $Q_{\mathrm{INC}}$ (line 21), releasing resources until all of the slices equally share the AP. Otherwise, the quantum value of the BE slices is decreased by a factor of $Q_{\mathrm{DEC}}$ (lines 14, 16, and 20), delaying such traffic from being dequeued. This leaves more resources for the QoS-constrained slices and the channel less busy for any QoS-constrained flows from STAs, favoring them to be satisfied (recall Section 3.1).
**Algorithm 2** Network Slicing Algorithm**Input:**
 1: every                  ▹ configuration loop interval (5 s used)
 2: $\forall s\in S^{b}:D_{\mathrm{QoS}}^{s}$               ▹ max queueing delay of each slice *s*
 3: $\forall s\in S^{b}:\mu_{\mathrm{QoS}}^{s}$                 ▹ min throughput of each slice *s*
 4: $\forall f\in F,\forall t\in T:\mu_{\mathrm{QoS}}^{f,t}$        ▹ min throughput of each flow *f* from STA *t*
 5: $Q_{\mathrm{MIN}}$, $Q_{\mathrm{MAX}}$             ▹ min, max quantum (10 us, 12,000 us used)
 6: $Q_{\mathrm{INC}}$, $Q_{\mathrm{DEC}}$             ▹ increase, decrease factors (10%, 90% used)


 7: **loop** 
every
 8:     **for each**
b∈B
**do**                     ▹ iterate over all APs
 9:         reconfigure(*b*,requirementsMet*(b)*)


 10:
 11: **function** 
requirementsMet(*b*)
 12:     **for each**
$s\in S^{b}$
**do**               ▹ iterate over all slices of an AP
 13:         **if**
$D_{\mathrm{QoS}}^{s}$
**then**
 14:           **if**
$D^{s}>D_{\mathrm{QoS}}^{s}$
**then**
**return**
$Q_{\mathrm{DEC}}$

 15:        **if**
$\mu_{\mathrm{QoS}}^{s}$
**then**
 16:            **if**
$\mu^{s}<\mu_{\mathrm{QoS}}^{s}$
**then**
**return**
$Q_{\mathrm{DEC}}$


 17:     **for each**
t∈T
**do**                    ▹ iterate over all STAs
 18:         **for each**
$f\in F^{t}$
**do**
 19:            **if**
$\mu_{\mathrm{QoS}}^{f,t}$
**and**
$t^{b}=true$
**then**
 20:                **if**
$\mu^{f,t}<\mu_{\mathrm{QoS}}^{f,t}$
**then**
**return**
$Q_{\mathrm{DEC}}$


 21:     **return**
$Q_{\mathrm{INC}}$

 22:
 23: **function** 
reconfigure($b,Q_{\mathrm{FACTOR}}$)
 24:     **for each**
$s\in S^{b}$
**do**               ▹ iterate over all slices of an AP
 25:         **if**
$(D_{\mathrm{QoS}}^{s}==\emptyset$
**and**
$\mu_{\mathrm{QoS}}^{s}==\emptyset )$
**then**
 26:            $Q^{s}\leftarrow$
getCurrentQuantum(*s*)
 27:            $Q_{\mathrm{NEW}}^{s}\leftarrow Q^{s}\;\cdot \;Q_{\mathrm{FACTOR}}$
 28:            **if**
$Q_{\mathrm{NEW}}^{s}>Q_{\mathrm{MAX}}$
**then**
$Q_{\mathrm{NEW}}^{s}\leftarrow Q_{\mathrm{MAX}}$
 29:            **if**
$Q_{\mathrm{NEW}}^{s}<Q_{\mathrm{MIN}}$
**then**
$Q_{\mathrm{NEW}}^{s}\leftarrow Q_{\mathrm{MIN}}$
 30:            **if**
$Q_{\mathrm{NEW}}^{s}\neq Q^{s}$
**then**
*b*.setSlice
$\left(Q_{\mathrm{NEW}}^{s}\right)$   ▹ set new slice quantum on AP


In the slicing algorithm, both throughput and queueing delay requirements are verified. For throughput, the algorithm checks the SMA of the last ten measurements. For queueing delay, the algorithm checks the SMM instead to avoid the masking effect in the presence of outliers. A new quantum $Q_{\mathrm{NEW}}^{s}$ is set for a slice on an AP only when it differs from its current one. $Q_{\mathrm{MIN}}$ and $Q_{\mathrm{MAX}}$ are thresholds that prevent traffic in BE slices from being blocked and from exceeding a maximum quantum configuration, respectively. Recall that, in the ADWRR scheduling algorithm, inactive traffic rules do not cause any performance degradation to the system. The ADWRR scheduling algorithm only iterates over active queues/slices. In addition, limiting the resources that a slice might utilize only occurs when multiple slices of an AP remain active and, therefore, must compete for access to the same NIC; otherwise, a slice may freely utilize all of the resources available.

### 4.4. Traffic Shaping Algorithm

Algorithm 3 is used for managing the traffic from STAs by shaping UL traffic. The traffic shaping adjustments are again based on three thresholds: the maximum queueing delay of the QoS slices, the minimum throughput of the QoS slices, and the minimum throughput of the QoS flows measured from the STAs. While using the same principle as Algorithm 2, the traffic shaping algorithm tries to satisfy the QoS requirements of the QoS flows in both DL and UL directions. By adjusting the traffic shaping configuration of STAs, which have BE flows only, the algorithm reduces the amount of traffic sent in favor of QoS-constrained traffic.

In each reconfiguration loop, the algorithm checks, for each AP, whether the requirements of all QoS slices and all QoS-constrained flows from the STAs associated with it are met. When all of the requirements of an AP are met, the traffic shaping configuration of the STAs, which have BE flows only and they are sharing the AP with a QoS-constrained flow from an STA or slice, is increased by a factor of $\lambda_{\mathrm{INC}}$ (line 21). Otherwise, the traffic shaping configuration is decreased by a factor of $\lambda_{\mathrm{DEC}}$ (line 14), reducing all of the traffic sent by those STAs, leaving the channel less busy for the QoS-constrained flows from STAs and slices.

Similar to Algorithm 2, throughput and queueing delay requirements are checked in order to ensure they are met; the SMA is used for throughput and the SMM for queueing delay. In addition, the algorithm verifies whether STAs have active flows, otherwise no reconfiguration is required. A new traffic shaping configuration $\lambda_{\mathrm{NEW}}^{s}$ is set for an STA, only when it differs from its current one. $\lambda_{\mathrm{MIN}}$ and $\lambda_{\mathrm{MAX}}$ are thresholds that prevent that traffic shaping configuration from exceeding minimum and maximum boundaries, which prevents STAs from having all connectivity blocked or from exceeding a maximum traffic shaping configuration.
**Algorithm 3** Traffic Shaping Algorithm**Input:**
 1: every                  ▹ configuration loop interval (5 s used)
 2: $\forall s\in S^{b}:D_{\mathrm{QoS}}^{s}$               ▹ max queueing delay of each slice *s*
 3: $\forall s\in S^{b}:\mu_{\mathrm{QoS}}^{s}$                 ▹ min throughput of each slice *s*
 4: $\forall f\in F,\forall t\in T:\mu_{\mathrm{QoS}}^{f,t}$        ▹ min throughput of each flow *f* from STA *t*
 5: $\lambda_{\mathrm{MIN}}$, $\lambda_{\mathrm{MAX}}$    ▹ min, max value for The traffic shaper. (1 Mbps, 100 Mbps used)
 6: $\lambda_{\mathrm{INC}}$, $\lambda_{\mathrm{DEC}}$             ▹ increase, decrease factors (10%, 90% used)


 7: **loop** 
every
 8:     **for each**
b∈B
**do**                     ▹ iterate over all APs
 9:         reconfigure(*b*,requirementsMet(*b*))


 10:
 11: **function** 
requirementsMet(*b*)
 12:     **for each**
$s\in S^{b}$
**do**               ▹ iterate over all slices of an AP
 13:         **if**
$D_{\mathrm{QoS}}^{s}$
**then**
 14:           **if**
$D^{s}>D_{\mathrm{QoS}}^{s}$
**then**
**return**
$\lambda_{\mathrm{DEC}}$

 15:        **if**
$\mu_{\mathrm{QoS}}^{s}$
**then**
 16:            **if**
$\mu^{s}<\mu_{\mathrm{QoS}}^{s}$
**then**
**return**
$\lambda_{\mathrm{DEC}}$


 17:     **for each**
t∈T
**do**                    ▹ iterate over all STAs
 18:         **for each**
$f\in F^{t}$
**do**
 19:            **if**
$\mu_{\mathrm{QoS}}^{f,t}$
**and**
$t^{b}=true$
**then**
 20:                **if**
$\mu^{f,t}<\mu_{\mathrm{QoS}}^{f,t}$
**then**
**return**
$\lambda_{\mathrm{DEC}}$


 21:     **return**
$\lambda_{\mathrm{INC}}$

 22:
 23: **function** 
reconfigure($b,\lambda_{\mathrm{FACTOR}}$)
 24:     **for each**
t∈T
**do**                    ▹ iterate over all STAs
 25:         **if**
$t^{b}=true$
**then**
 26:            **for each**
$f\in F^{t}$
**do**
 27:                **if all**(f==∅
**for**
*f* in $\mu_{\mathrm{QoS}}^{f,t})$
**and**
getActiveFlows(*t*) **then**
 28:                    $\lambda^{t}\leftarrow$
getCurrentTrafficShaper(*t*)
 29:                    $\lambda_{\mathrm{NEW}}^{t}\leftarrow \lambda^{t}\;\cdot \;\lambda_{\mathrm{FACTOR}}$
 30:                    **if**
$\lambda_{\mathrm{NEW}}^{t}>\lambda_{\mathrm{MAX}}$
**then**
$\lambda_{\mathrm{NEW}}^{t}\leftarrow \lambda_{\mathrm{MAX}}$
 31:                    **if**
$\lambda_{\mathrm{NEW}}^{t}<\lambda_{\mathrm{MIN}}$
**then**
$\lambda_{\mathrm{NEW}}^{t}\leftarrow \lambda_{\mathrm{MIN}}$
 32:                    **if**
$\lambda_{\mathrm{NEW}}^{t}\neq \lambda^{t}$
**then**
$t.setTrafficShaper(\lambda_{\mathrm{NEW}}^{t})$▹ set traffic shaping on STA


## 5. Evaluation

**Figure 6:**
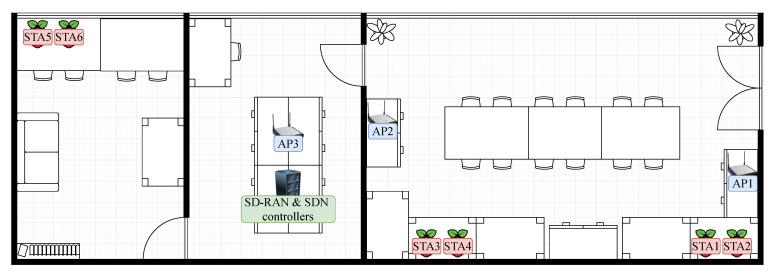
Testbed deployment scenario.

In this section, we present the evaluation of our approach in a real-world testbed. Figure 6 depicts the layout of the testbed. The setup is made up of a single computer hosting the SD-RAN and backhaul controllers, three APs, and six STAs. The computer hosting the controllers is connected to the wired segment as well as the APs. The APs are based on the PC Engines APU2D4 (x64) processing board, equipped with one Qualcomm Atheros AR958x 802.11 a/b/g/n each. The STAs are Raspberry Pis 4 Model B+ with 802.11b/g/n/ac.

In our setup, APs and STAs are positioned in three rooms, including two offices that are separated by a server room. We have set the APs to operate on non-overlapping channels, specifically on channels 1, 6, and 11 for APs 1, 2, and 3, respectively. The supported Modulation and Coding Scheme (MCS) rate indices are from 0 to 7, because the STAs operate in the 2.4 GHz band. Our experiments were conducted in a closed office environment with little to no external interference. With this setup, we evaluate whether QoS delivery can be enhanced targeting MCAs, such as process automation and remote control. Although our setup is built in an office environment, in future digital factories, production lines are usually confined to specific locations under private ownership; therefore, the level of interference in such private spaces can also be controlled.

Process automation and remote control encompasses the use case scenario for the automation of reactive flows requiring low latency and high service availability (e.g., refineries and water distribution networks). Within digital factories, some of the interactions among components are conducted by automated control applications. The monitoring and management of distributed control systems usually takes place in a dedicated control room and there is the need for controlling real-time data provided to the control room, by the local staff. For such MCAs, the typical end-to-end latency expected is to be around 50 ms and the user experienced data rates, communication service availability, and connection density may vary. According to [2], while the staff on location needs to view inaccessible locations (e.g., emergency valves) with high definition, the personnel in the control room benefit from high-definition footage (High-Definition (HD) or even 4K) from body cameras. Thus, these applications require data rates that range from 1 Mbps to 100 Mbps. In our experimentation, we run services where the queueing delay and throughput requirements are similar to these expected boundaries. Queueing at the RAN often presents bottlenecks when resources becomes scarce, which makes it essential to avoid such bottlenecks that degrade the performance of MCAs.

We generate several User Datagram Protocol (UDP) flows, in which each flow represents a different service in the network. For each DL flow, the SD-RAN controller creates a dedicated slice with the default quantum $Q^{s}$ of 12,000 us on the AP in which the STA is receiving such a flow. We have set equal quantum configurations to slices in order to verify whether MCA application requirements can be satisfied when resources are equally distributed, hence verifying the need for performing airtime-based network slicing at runtime. The backhaul controller is instructed by the SD-RAN controller to install the corresponding traffic rules (i.e., OpenFlow rules) and map flows into slices. On the other hand, for each of the UL flows, the SD-RAN controller sets an initial traffic shaping configuration $\lambda^{t}$ of 100 Mbps to all STAs. This value was chosen to bootstrap the traffic shaping configuration while not compromising the throughput of STAs in advance. The traffic is generated between the computer hosting the controllers and the STAs in both DL and UL directions. In order to avoid static flow rates and arrival times, we generated the flows following the Poisson distribution with MGEN (https://www.nrl.navy.mil/itd/ncs/products/mgen), a toolset for generating real-time traffic patterns, having a fixed frame size of 1024 bytes. The parameters used for the user association, network slicing, and traffic shaping algorithms are given in Algorithms 1–3. For the evaluation, we created the following three experimental setups:

- Experiment 1: we evaluate four different scenarios to show how our network slicing and traffic shaping algorithms can provide enhanced QoS delivery when flows of different priorities classes (BE and QoS) and in different directions have to compete with one another. These scenarios were run for five minutes each, with only 200 s presented.
- Experiment 2: we compare the performance of our approach to a state-of-the-art user association approach from Gómez et al. [5]. We run flows in the DL direction and analyze whether our approach can enhance the QoS delivery of the QoS-restricted slices dedicated to such flows, at runtime. We analyze whether the QoS requirements for throughput and queueing delay can be maintained along the experiment run. This experiment was run for ten minutes.
- Experiment 3: we analyze whether our whole system can enhance QoS delivery, again in comparison to the approach from Gómez et al. We run flows in both directions and analyze whether the QoS requirements for throughput and queueing delay of slices can be maintained along the experiment run. This experiment was run for ten minutes.

In this paper, we assume no QoS differentiation among QoS services themselves, especially when they compete for resources on the same AP. In such cases, as we consider that they belong to the same group of MCAs and, therefore, are equally important. Thus, our premise is to provide them an equal amount of resources. As defined, the ADWRR scheduling algorithm does not allow for limiting the maximum throughput of slices; this problem requires a new implementation and it is part of our future work. Although QoS differentiation could be addressed with an intra-AP perspective, when multiple APs are sharing a channel and have conflicting QoS requirements, the problem is complex and often without a feasible solution. Because of this, we consider this problem outside the scope of our work. Our evaluation focuses on identifying whether the queues on APs represent bottlenecks that compromise the QoS required by MCAs. We also evaluate whether our approach is capable of enhancing the QoS delivery of such MCAs at runtime, under high, but feasible, demands. In order to prevent QoS slices from overuse of resources, we assume that any SDN-based admission control system can be introduced as necessary.

### 5.1. Experiment 1: Traffic Shaping and Airtime-Based Network Slicing

In this experiment, we evaluate whether our solution can satisfy the QoS requirements when flows of different priority classes (BE and QoS) and in different directions (UL and DL) are served by a single AP. For this purpose, we used a single AP and two STAs to provide a better understanding of how our solution acts and what are the obtained results. Specifically, we use STAs 1 and 2 connected to AP 1 for this first experiment. Table 5 contains the different scenarios that we evaluate.

#### 5.1.1. Scenario A: UL BE versus UL QoS

**Figure 7:**
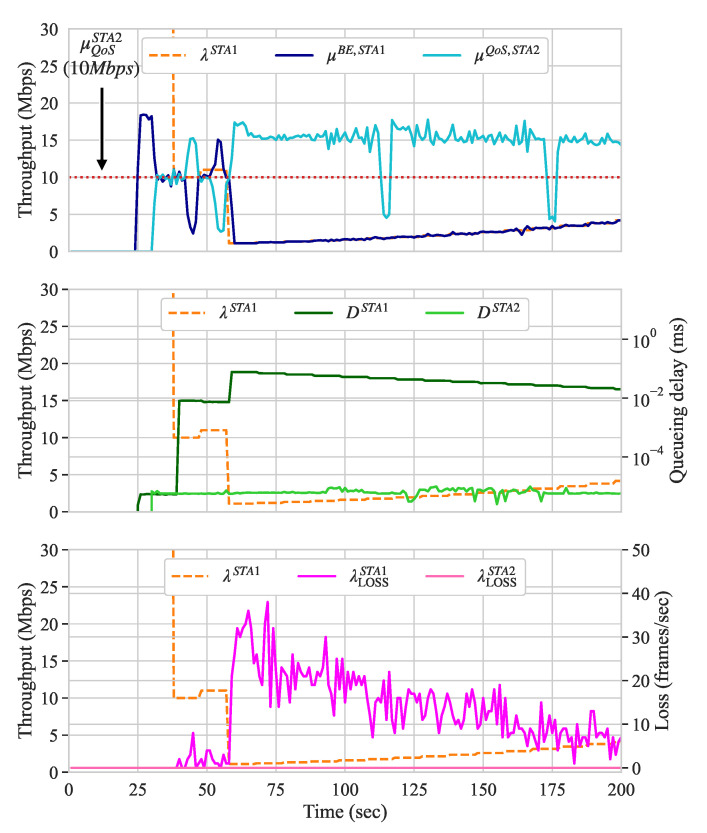
Scenario A.

Figure 7 presents the throughput, queueing delay, and frame loss, along with the traffic shaping configurations for scenario A. In this scenario, two UL flows are competing for the channel: a BE flow originating from STA 1 and a QoS flow originating from STA 2. The QoS flow requires a guarantee of 10 Mbps of throughput.

In order to highlight the need for traffic shaping, we start both flows and, only after a few seconds (at second 25), we activate our approach to verify whether QoS requirements are met or not. Recall that, to verify the throughput requirements, the controller considers the SMA of the last ten measurements, i.e., the SMA of the last ten measurements. Because the throughput of the QoS flow was, on average, below its QoS requirements (i.e., below 10 Mbps), the traffic shaping configuration of STA 2 transmitting the BE flow is decreased. The increase and decrease rates used correspond to 10% and 90% of its current value, while the reconfiguration frequency is set to ten seconds.

As expected, the traffic shaping configuration of STA 2 continues to decrease until the QoS requirements of flows and slices served by the AP are met or its configuration reaches the $\lambda_{\mathrm{MIN}}$ of 1 Mbps. As we see, both the queueing delay and frame loss of STA 2 increase when the traffic shaping configuration is below its throughput. If all the QoS requirements are met, the traffic shaping configuration is gradually increased until it reaches the $\lambda_{\mathrm{MAX}}$ of 100 Mbps. While such traffic shaping imposes frame loss and an increase on the queuing delay of STA 1 originating the BE flow, it allows for the QoS flow to better maintain its throughput requirement.

#### 5.1.2. Scenario B: UL BE versus DL QoS

**Figure 8:**
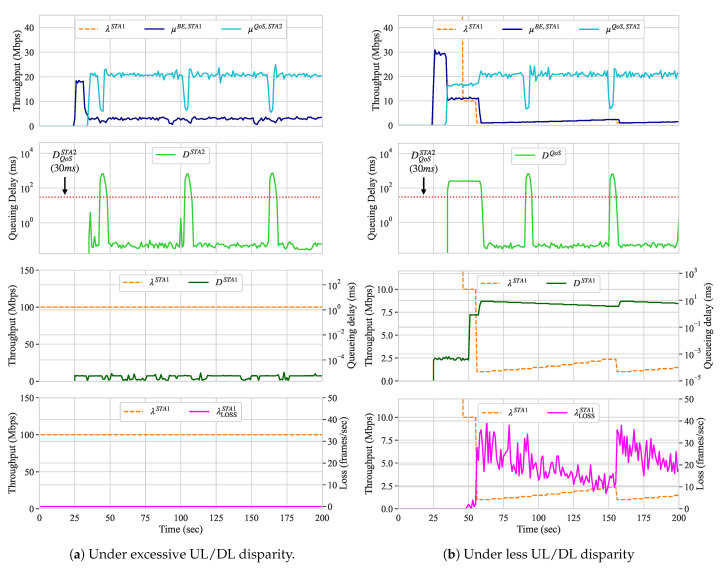
Scenario B.

In this scenario, we introduce a BE flow in the UL direction at STA 1 to compete against a QoS flow, handled by a QoS slice, in the DL direction at STA 2. The queueing delay of the QoS slice should be less than 30 ms. In this scenario, we expect shaping to be activated on STA 1 in order to fulfill the QoS requirements of STA 2. However, even after increasing the throughput demand of STA 2 to 20 Mbps, the results show that the QoS requirements were met without traffic shaping. We believe this is because IEEE 802.11 networks are known to have unfairness between UL and DL accesses under the Distributed Coordination Function (DCF) [63] and, in this scenario, the UL/DL throughput was unbalanced. This favored the queueing delay requirements of the QoS slice to be satisfied, against our expectations. Figure 8a presents the throughput, traffic shaping configuration, queueing delay, and frame loss of STA 1 (the STA that originates the BE flow) along with the throughput and queueing delay of the QoS slice handling the traffic towards the controller (through the AP), while using the original Raspberry Pi node.

We swapped STA 1 with a node capable of transmitting frames using higher MCSs indexes similar to the transmission capabilities of the AP in order to confirm our belief and demonstrate the traffic shaping in this scenario. For this, we used a PC Engines APU2C4 node that was equipped with Qualcomm Atheros QCA986x/988x 802.11b/g/n/ac. Thus, the UL flow originating from this node comprises a QoS slice and traffic shaping is needed. Figure 8b presents the results for this scenario with the more capable node. In Figure 8a, although $D_{\mathrm{QoS}}^{STA2}$ was not met for short intervals (≈ 5 s) due to the Address Resolution Protocol (ARP) messages, Algorithm 3 uses the SMM of the last ten measurements to indicate whether such requirements are met or not. If the reconfiguration loop does not coincide with the periods where the SMM indicates that the QoS requirements for the queueing delay are met, the traffic shaping configuration is not decreased.

After the QoS slice starts dequeueing its frames and, therefore, competes for the channel with the UL flow, traffic shaping at STA 1 is applied. Around second 50, the traffic shaping configuration starts to decrease and, as a result, the queueing delay and frame loss for the UL flow increase accordingly, as Figure 8b shows. When the QoS is met, the value of the traffic shaping configuration is increased and both queueing delay and frame loss decrease until the configuration reaches the $\lambda_{\mathrm{MAX}}$ of 100 Mbps. When the queueing delay of the QoS slice is affected by the ARP messages and the reconfiguration loop gets triggered (at around second 155), the traffic shaping configuration decreases once again. As a consequence of such spikes on both queueing delay and throughput measurements, in our approach, one can set different parameters to he measurement window and, therefore, consider those to be outliers. However, as a trade-off, several additional samples are required in order to identify that network re-configurations have impacted the measurements. In this manner, in addition to higher or lower adaptation rates, one can set the size of the measurement window resulting in a more steady or loose behavior for the BE flows.

#### 5.1.3. Scenario C: DL BE versus UL QoS

**Figure 9:**
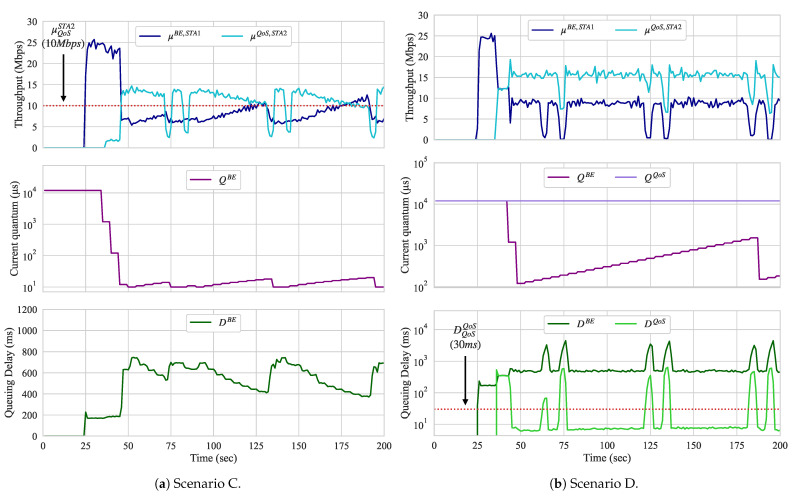
Scenario C and D.

Until now, only traffic shaping was performed. In this scenario, we run an UL flow with QoS requirements that competes with a BE flow that is handled by a BE slice. In this case, actions upon the BE slice are required. Because the ADWRR does not provide the means to limit the maximum throughput of slices, the traffic flowing through a slice can be either delayed by decreasing its quantum configuration, or blocked by assigning a non-positive value to its quantum configuration. We set the parameters of Algorithm 2 to not block The entire flow of any slice, but to attempt to introduce delay by reducing its quantum configurations. Figure 9a presents the throughput of the UL QoS flow and the throughput, queueing delay, and quantum configuration of the DL BE slice.

While the load of the UL flow is 15 Mbps, the QoS throughput threshold is only 10 Mbps. As we see when the QoS flow starts (at around second 40), its throughput is less than half of what is required. Therefore, Algorithm 2 performs quantum adjustments on the BE slice running on the AP. The algorithm starts to decrease the quantum configuration until all QoS requirements are met or its value reaches the $Q_{\mathrm{MIN}}$ of 10 us. When the 10 Mbps throughput is met, the quantum is gradually increased and, as a result, frames are dequeued at a faster pace and queueing delay reduces. When QoS is met, the quantum configuration increases instead with an upper bound $Q_{\mathrm{MAX}}$ of 12,000 us. The increase and decrease rates used for the quantum adaptation correspond to 10% and 90% of its current value, as presented in Algorithm 2.

#### 5.1.4. Scenario D: DL BE Versus DL QoS

In this scenario, two slices of different priority classes (BE and QoS) compete for resources on the same AP. The QoS slice requires that its queueing delay remains less than 30 ms for a throughput demand of 15 Mbps. Figure 9b presents the throughput, queueing delay, and quantum configuration of both the BE and QoS slices.

As we can observe, after the QoS slice starts dequeueing its flow, both BE and QoS slices compete for the resources of the AP. At this time, because slices have the same quantum configuration, both STAs receiving such flows experience similar channel conditions and the data rates used for their transmissions are similar. Both throughput and queueing delay are also similar; however, the queueing delay experienced by both corresponds to almost 400 ms each. Therefore, Algorithm 2 is activated and the quantum of BE slice starts to adjust around second 40. When the QoS requirements of the QoS slice are not met, the quantum configuration for the BE slice is decreased until the QoS requirements are met or the quantum configuration of the BE slice reaches the $Q_{\mathrm{MIN}}$ of 10 us. When the QoS requirements are satisfied, the quantum configuration of the BE slice is gradually increased until the QoS of the QoS is satisfied or its value reaches the $Q_{\mathrm{MAX}}$ of 12,000 us.

### 5.2. Experiment 2: DL QoS Delivery and User Association

In experiment 2, we evaluate whether our approach can enhance the QoS delivered when the network only has DL flows. Recall that, for each DL flow, a dedicated slice is created in order to handle the flow. In this case, only network slicing is performed, with no traffic shaping occurring on the STAs, although we expect our solution to perform MCDA-based handovers when necessary. We compare our approach to the user-association algorithm from Gómez et al. Their approach uses the average RSSI of an AP, AP load, and channel occupancy to determine which AP is ideal for a given STA. In addition to those factors, our approach considers the queueing delay of slices, the expected load on APs, and the association status of STAs. Table 6 presents the workload parameters used in experiment 2. We start each experiment with The following initial STA/AP association: STAs 1 and 2 are associated with AP 1, STAs 3 and 4 with AP 2, and STAs 5 and 6 with AP 3. The experiment was run for a total of ten minutes, with four events occurring within the first third of the experiment. This was done in order to show the performance of the system during a period, where the network demand varies, as well as a more consistent, less active period.

**Figure 10:**
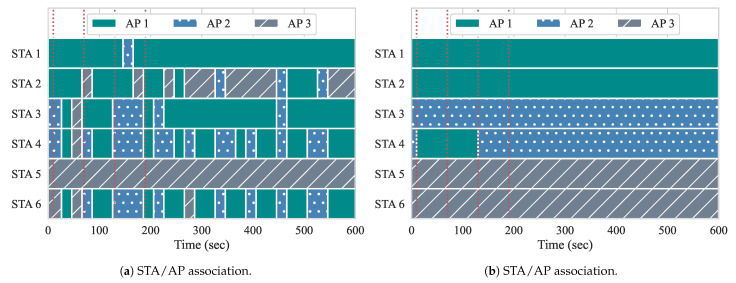
Experiment 2 association. (**a**) Gómez et al. [5]; (**b**) Proposed.

Figure 10 shows the association of the STAs among the three APs throughout the duration of the experiment, while using both of the approaches. The red vertical dotted lines illustrate when the events occur. There are no flows active at the beginning of the experiment, so, with the approach from Gómez et al., the ideal AP is determined by overall channel load and RSSIs. Additionally, since there are no active flows in the network and handovers at this point might be unnecessary and cause overhead to APs and controller, our approach does not compute any user re-association. For both of the experiments, two STAs are associated with each AP to begin the experiment.

At event 1, two DL flows of 20 Mbps each (BE flows 3 and 4) are started from the controller to STAs 3 and 4, respectively. Handovers begin to occur with the first iteration of the reconfiguration loop at second 20. As we can observe, both of the approaches perform their first handovers. With yhe approach from Gómez et al., STAs 3 and 4 are moved from AP 2 to AP 1, as well as STA 6 from AP 3 to AP 1. The algorithm computed that the channel load of AP 1 in conjunction with the RSSI gathered from these STAs is favorable, and, therefore, triggered the handovers accordingly. Because there is no flow active at AP 3, STA 6 most likely moved based on the average RSSI. However, with the next reconfiguration, STAs are handed off to AP 3, since the AP load at AP 1 is now high. This is an example of the algorithm from Gómez et al. experiencing the ping-pong effect. In contrast, since our approach allows for one handover per AP pair at each reconfiguration loop, only STA 4 suffers a handover as a result of event 1. Once it is moved to AP 1, APs 1 and 2 experience similar loads, while AP 3 remains free until the next events occur. Uneven loads, such as this, can occur, because only two STAs are being served, while three APs are available. This can lead to the ping-pong effect. as we can observe with Gómez et al. With our approach, the ping-pong effect does not occur, as it considers the association status of STAs and expected load of APs.

Event 2 occurs at second 70, and introduces two more BE flows, this time to STAs 2 and 5. With our approach, as the load of APs are similar, so this event does not require handovers. With the approach from Gómez et al., STA 5 does not suffer a handover, while the rest of the STAs move quite frequently. We believe that the reason for this behavior is because the RSSIs perceived for APs 1 and 2 are either not favorable, or unavailable. We analyze the RSSI measurements in Section 5.2.1. Although, in our scenario, all STAs are reachable and perceived by all APs, this might not always be the case. When STAs are not perceived by all APs, the alternatives APs are filtered out according to whether the average RSSIs, perceived from STAs, can be computed or not. When we run the approach from Gómez et al., STAs only perform a handover if the selected AP has measured RSSIs. When there are no measurements regarding the RSSI, the algorithm assumes that the STA is not within range for the APs, so handovers to those are not considered.

At event 3, we can see that Gómez et al. performs three handovers, moving STAs 3, 4, and 6 to AP 2. This is appropriate, as AP 2 had the lowest load of all three APs. Our algorithm also moves STA 3 back to AP 2. At this point, there are only active flows going to STAs 2 and 5, which are associated with separate APs in our approach. At event 4, the first QoS flows are introduced, going to STAs 1 and 6, with loads of 10 Mbps and 8 Mbps, respectively. In our approach, neither STA 1 nor STA 6 suffer a handover after event 4. In fact, neither STA suffers a handover for the duration of the experiment. Meanwhile, with Gómez et al., while STA 1 does not suffer handovers after event 4, STA 6 suffers 12 handovers, while it is receiving a QoS flow. This negatively impacts its QoS requirements, as handovers require the STA to change channels when switching APs, resulting in more downtime, less throughput, and higher delay. Additionally, the Gómez et al. approach also performs many handovers of STAs 2 and 4 after event 4, which leads to more performance degradation among BE flows as well. Overall, our approach only performed two handovers, while the Gómez et al. approach performed 60 handovers, since it suffered from the ping-pong effect. This impacted both queueing delay and throughput when running both of the approaches.

**Figure 11:**
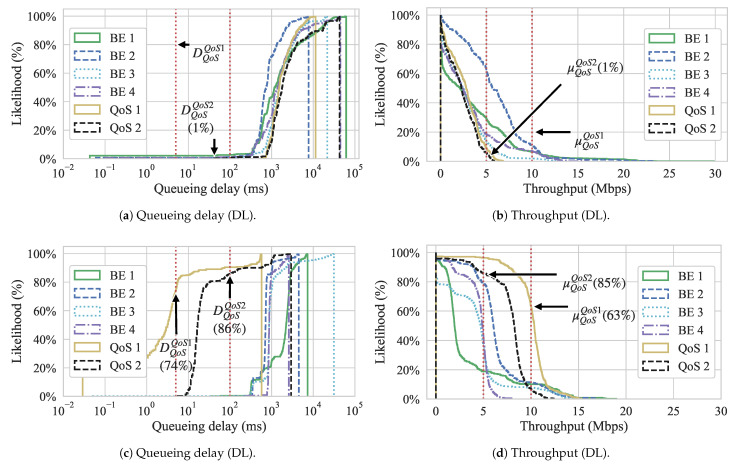
Experiment 2 Cumulative Distribution Functions (CDFs). (**a**) Queueing and (**b**) throughput of slices (DL) with Gómez et al. [5]; (**c**) queueing delay; and. (**d**) throughput of slices (DL) with our approach.

Figure 11 shows the Cumulative Distribution Function (CDF) graphs for both queueing delay and throughput for both approaches. These graphs show the likelihood of achieving a certain throughput or queueing delay during the experiment. The red vertical dotted lines show the QoS requirements for the QoS flows. Regarding throughput, since we are interested in showing the likelihood of flows having higher value occurrences, we show the inverse CDF. We can observe, from Figure 11a,b, that flow QoS 1 never reached its QoS requirement, while QoS flow 2 has less than 1% chance of meeting its requirement for the Gómez et al. approach. On the other hand, looking at Figure 11c, we can see that, with our approach, the same flows have a 74% and 86% chance to meet the queueing delay of QoS flow 1 and 2 while the throughput is met with a 63% and 85% probability for QoS 1 and 2, respectively.

**Figure 12:**
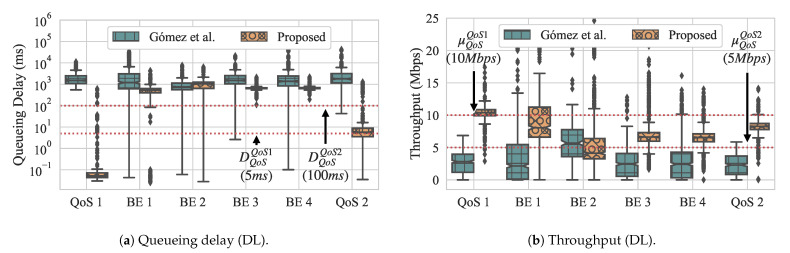
Experiment 2 box-and-whiskers plots. (**a**) Overall queueing delay of slices (DL); and, (**b**) overall throughput of slices (DL).

Figure 12 presents the overall throughput and queueing delay while using box-and-whiskers plots for both approaches, with Figure 12a presenting the queueing delay and Figure 12b presenting throughput. Looking first at the results that were obtained with Gómez et al., we observe that the overall throughput for flow QoS 1 fails to meet its QoS requirement and flow QoS 2 only the lower whisker meets its QoS requirement. In contrast, in our approach, the upper quartile meets the requirement for flow QoS 1, and all of the quartiles meet the requirement for flow QoS 2 with only outliers from periodic spikes that are caused by control messages lying above the requirement. In other words, most of the time our approach is able to achieve the desired QoS requirements. For BE flows, our approach generally has much tighter variation among delay for its flows, while the delay for Gómez et al. varies highly.

For the dequeueuing rate, we can see that our approach yields higher throughput for both QoS flows, although, for QoS 1, the median lies at the requirement, which means that it does not fully satisfy the requirement all of the time. In contrast, Gómez et al. does not come close to meeting the QoS requirement for the QoS flow 1, with its median throughput around 3.5 Mbps. For flow QoS 2, only the upper whisker meets the throughput requirement. Similar to queueing delay, the throughput of the BE flows while using our approach are much tighter, with slightly higher medians. Using Gómez et al., the throughput of the BE flows have a higher variation and slightly lower medians.

#### 5.2.1. RSSI and User Association

**Figure 13:**
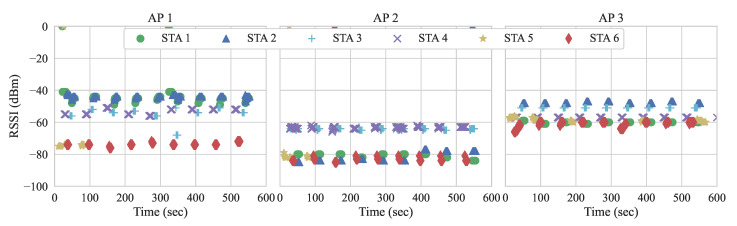
Received Signal Strength Indicator (RSSI) of STAs per AP with an initial STA/AP association.

In order to better understand the user association behavior in our setup, we monitor the RSSI perceived from STAs after their initial association with the APs. We collect the results for a period of ten minutes, where only ARP-related messages are flowing. Figure 13 present the RSSI measurements of STAs during the experiment run.

**Figure 14:**
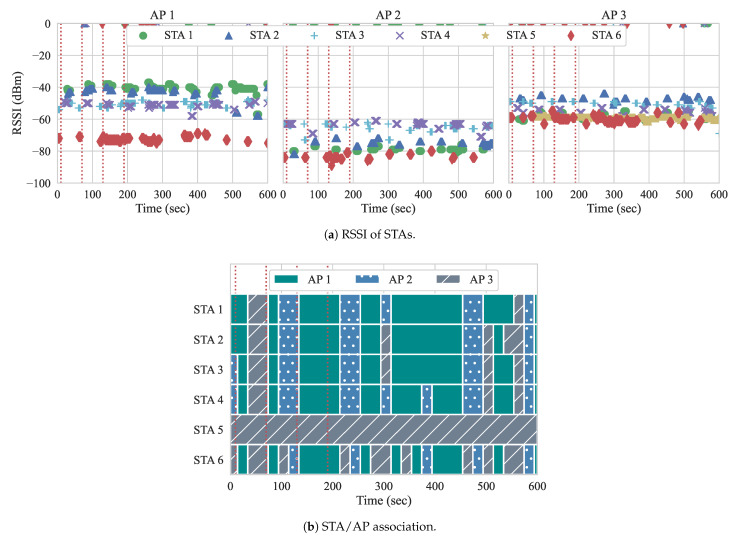
RSSI of STAs per AP running the workload of experiment 2 with Gómez et al. [5].

We observe that STA 5 is rarely perceived by APs 1 and 2, only when RSSIs are slightly higher than usual. This explains why STA 5 tends to be connected to AP 3. When RSSIs are not perceived by an AP, such an AP is not considered in subsequent handover decisions. For example, in some scenarios, not all STAs are within the range of APs and, therefore, must be filtered out. Nonetheless, when RSSIs are present, both of the approaches can apply their methods and consider such APs as alternatives for handovers. Given that the approach by Gómez et al. performed the most handovers in experiment 2, we run it again with the goal of verifying the user association with the RSSI measurements. Figure 14 shows the RSSI measurements and the association status running Gómez et al. with the workload presented in Table 6. The red vertical dotted lines illustrate when the events occur.

In Figure 14a, we observe that the RSSIs are similar to our previous run when only the traffic regarding ARP is flowing. However, as a consequence of the flows that were introduced by the events (Table 6), we observe a higher number of RSSI measurements. In this experimental run, AP 2 presents lower RSSIs in general, while AP 1 and AP 3 divide the best alternative AP according to this single criterion. Besides, we see that there are no RSSI measurements for STA 5 at APs 1 and 2. Therefore, in Figure 14b, STA 5 associates with AP 3 for the entire duration of the experiment.

### 5.3. Experiment 3: UL/DL QoS Delivery and User Association

For experiment 3, we have four similar events that occur in the first third of our ten-minute experiment. Now, we introduce BE and QoS flows in the UL direction. Table 7 presents the workload parameters that were used in this experiment. After the flows start, they run for the duration of the experiment, although BE flows 3 and 4 stop for a minute at event 3. As in the previous experimentation, we start each experiment with the following initial STA/AP association: STAs 1 and 2 are associated with AP 1, STAs 3 and 4 with AP 2, and STAs 5 and 6 with AP 3. In this way, we can verify whether the QoS requirements can be maintained throughout the duration of the experiment.

**Figure 15:**
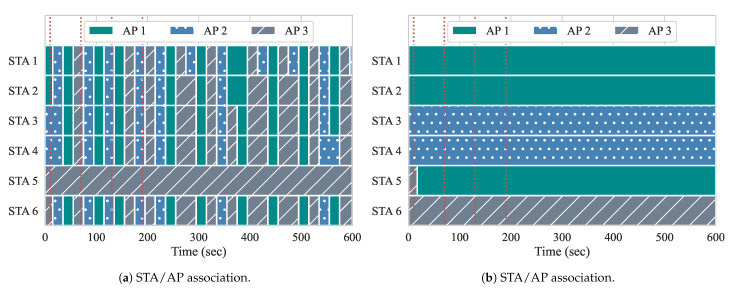
Experiment 3 association. (**a**) Gómez et al. [5]; (**b**) Proposed.

Similar to experiment 2, we present the STA–AP associations throughout this experiment in Figure 15. Here, we can see a stark contrast between the two approaches. With our approach, only a single handover is performed, while, with Gómez et al., 128 handovers are performed. Although our approach makes use of queueing delay and the expected load of flows to decide upon handovers, the results clearly show the need for considering the cost of a handover to prevent the ping-pong effect. With our approach, only a single handover per AP pair is allowed to be performed at each reconfiguration loop. The lone handover occurs just after event 1, when two DL BE flows start from the controller to STAs 5 and 6 and, in this case, are flowing through the same AP. i.e., AP 3. Our algorithm separates these two flows onto two separate APs in order to achieve enhanced throughput results.

On the other hand, when running the approach from Gómez et al., most of the handovers throughout are caused by the ping-pong effect. One notable detail is that STA 5 does not suffer a handovers during the experiment with Gómez et al., and this happens due to the same reason, as discussed in experiment 2. In short, RSSIs froth STA 5 are mostly not favorable or not available on APs 1 and 2.

**Figure 16:**
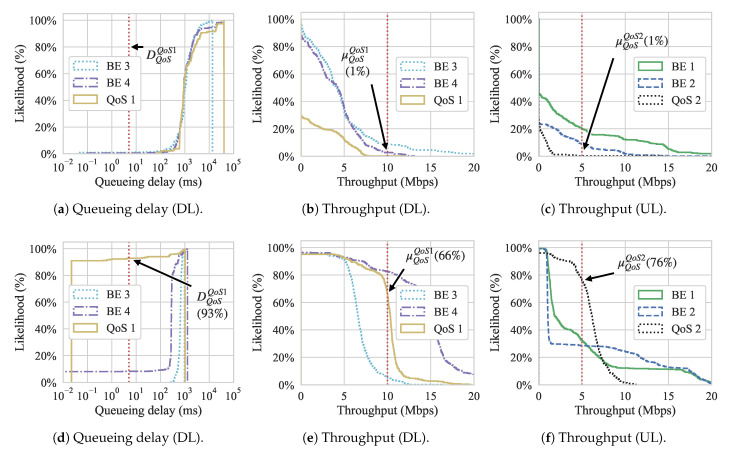
Experiment 3 CDFs. (**a**) Queueing delay of slices (DL), (**b**) throughput of slices (DL), and (**c**) throughput of flows from STAs (UL) with Gómez et al. [5]; (**d**) queueing delay of slices (DL), (**e**) throughput of slices (DL), and (**f**) throughput of flows from STAs (UL) with our approach.

In Figure 16, we present the CDF results for both throughput and queueing delay of slices on the APs, as well as the throughput measured from the UL flows. Again, the red vertical dotted lines show the requirements for the QoS flows. With the approach for Gómez et al., apart from an outlier for the throughput, both throughput and queueing delay requirements are never met. Meanwhile, our approach achieves the QoS requirements during most of the experimental duration. For the DL flow (QoS flow 1), the QoS requirements for both queueing delay and throughput of 5 ms and 10 Mbps are met with 93% and 66% of likelihood, respectively. For the throughput of the UL flow (QoS flow 2) is met for 76% of its active period.

**Figure 17:**
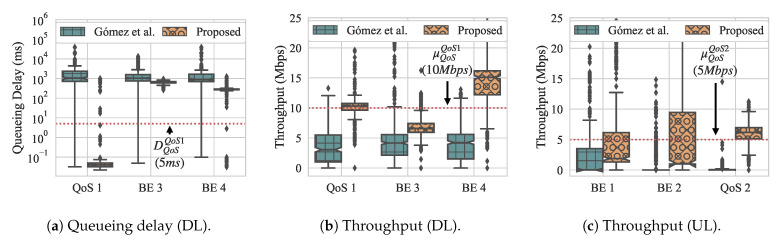
Experiment 3 box-and-whiskers plots. (**a**) Queueing delay of slices (DL); (**b**) throughput of slices (DL); (**c** throughput of flows from STAs (UL).

Figure 17 presents the overall throughput and queueing delay of slices and the overall throughput measured from the UL flows as box-and-whiskers plots. We can see that Gómez et al. achieves approximately equal delay for all flows, with much higher variability for BE flows, but it does not satisfy the QoS requirements. Only the bottom whisker lies below the delay threshold. On the other hand, with our approach, delay is achieved most of the time, with all three quartiles as well as the maximum and minimum whiskers lying below the threshold for QoS flow 1, and the median just about the threshold for QoS flow 2. There are outliers in our approach for both of the flows that do not meet the requirements. Similarly, for throughput, Gómez et al. has trouble meeting the QoS requirements for either QoS flow, while our approach achieves them (except for some outliers) for QoS flow 2, and achieves a median at the QoS threshold for QoS flow 1. As with experiment 2, our approach achieves much tighter quartiles for both delay and throughput.

## 6. Conclusions and Future Work

In this new era of 5G communication, MCAs are imposing stricter QoS requirements on coexisting technologies, such as IEEE 802.11 networks. Because traditional IEEE 802.11 networks are known to achieve insufficient reliability and non-deterministic latency, dynamic, and precise RA mechanisms are essential. In this paper, we proposed a delay-aware approach for MAC management via airtime-based network slicing and traffic shaping, as well as user association while using MCDA in IEEE 802.11 SD-RANs. With the centralized view of the network, our approach performs traffic shaping on the STAs to prevent BE flows from degrading the QoS requirements of others. Differently from most work in the literature, our approach considers QoS and BE flows in both the UL and DL directions.

Focusing on MCAs, we designed our experiments based on the QoS requirements from the use case of process automation and remote control in future digital factories. Through experimentation in a real-world testbed, our results show that our approach enhances the QoS support at runtime, drawing closer to the MCA requirements, as compared to a state-of-the-art user association algorithm [5].

As future work, we plan to address the design of a more deterministic scheduling algorithms for precise QoS service differentiation on the AP, the design of decentralized control algorithms for airtime-shaping and local decision-making, and the use of monitoring information with finer granularity for network management in general.

## Figures and Tables

**Table 1:** Summary of the main network slicing proposals in the IEEE 802.11 context.

Target	Resource Allocation/Isolation Method		Evaluation	Ref.
	UL	DL		
Airtime fairness via slicing	EDCA parameters	None	Simulation in Matlab	[34]
Airtime control via slicing		EDCA parameters	Simulation in QualNet	[35]
		Slice scheduling and traffic shaping	Testbed experimentation	[36]
		None	Simulation in Matlab	[37]
	None	Slice scheduling shaping		[20]
Experiment isolation in testbed	Slice scheduling	Slice scheduling	Testbed experimentation	[38]
Traffic isolation in testbed	Traffic shaping	None		[39]
Experimentation coexistence in testbed	None	Traffic shaping		[40]
Throughput guarantees		Slice scheduling		[41]
STA virtualization in testbed				[42]
Airtime control and traffic isolation via slicing			Simulation in NS3	[14]
	
				[15]
	
	Slice scheduling (indirect)			[16]

			Testbed experimentation	[4]

Airtime policy enforcement mechanism	None			[17]

Adaptive airtime-based slice orchestration				[18]

Optimal airtime-based RA modelling for network slicing			Testbed experimentation and theoretical analysis	[19]



**Table 2:** Summary of the main centralized-horizontal user association and load balancing solutions in Software-Defined Networking (SDN)-based IEEE 802.11 networks.

Main Target	Input Parameters/Metrics	Evaluation	Ref.
Support high-density AP deployment	RSSIs from both STAs and APs	Testbed experimentation	[44]
Customization and control of high-level policies	RSSIs, packets/bytes counters, airtime utilization, transmission failures, and re-transmissions		[45]
Mobility support and throughput enhancements	RSSIs, AP load, STA/AP distance, and STAs’ assignment status		[53]
	RSSIs, AP load, location, and STAs’ assignment status		[54]
	Users’ activity time and SNR of beacons and probe requests		[49]
	RSSIs and load of APs		[47]
	SNR of probe requests and APs’ channel utilization		[51]
	RSSIs, average load of APs, and average channel occupancy		[3]
	Average RSSIs, average load of APs, and average channel occupancy		[5]
	RSSI threshold		[46]
		OMNeT++ simulation	[48]
Load balancing, QoS and QoE support	SINR, bandwidth, jitter, and delay	OPNET simulation	[52]
Mobility support and multicast	Video quality, user demand, and RSSI of beacons	Testbed experimentation and simulation	[50]
	AP load, STAs’ SNR, and throughput requirements		[55]

**Table 3:** Overview of notation used.

Symbol	Description
*n*	The number of services to be delivered.
*B*	The set of APs of The network.
$S^{b}$	The set of slices of AP, b∈B.
*T*	Set of STAs of The network.
$t^{b}$	True if STA t∈T is associated with AP b∈B.
$t_{\mathrm{RSSI}}^{b}$	The measured RSSI from STA *t* on AP *b*.
$F^{t}$	The set of flows measured from STA *t*.
*f*	A flow measured from an STA, $f\in F^{t}$.
$\theta^{b}$	The overall channel load of AP *b*.
$D^{b}$, $D^{s}$, $D^{t}$	The measured queueing delay of AP *b*, of slice *s*, and of STA *t*.
$D_{\mathrm{QoS}}^{s}$	The maximum queueing delay threshold of slice *s*.
$\mu^{b}$	The measured throughput of AP *b*.
$\mu^{s}$	The measured throughput of slice *s*.
$\mu^{f,t}$	The throughput of flow *f* measured from STA *t*.
$\mu_{\mathrm{QoS}}^{s}$	The minimum throughput threshold of slice *s*.
$\mu_{\mathrm{QoS}}^{f,t}$	The minimum throughput threshold for flow *f* measured from STA *t*.
$\mu_{\mathrm{EXP}}^{b}$, $\mu_{\mathrm{EXP}}^{t}$	The overall expected throughput of AP *b*, and from STA *t*.
$\mu_{\mathrm{EXP}}^{s,t}$, $\mu_{\mathrm{EXP}}^{f,t}$	The expected throughput for STA *t* in slice *s*, and of flow *f* from STA *t*.
C	The set of MCDA criteria.
$W_{\mathrm{BE}}$,$W_{\mathrm{QoS}}$	The set of MCDA weights used for The BE and QoS flows.
$W^{t}$	The set of MCDA weights used for *t*.
$b_{\mathrm{STATS}}$	The set of monitoring statistics of *b*.
$b_{\mathrm{BEST}}$	The highest-ranked AP *b* of a given STA. $b_{\mathrm{BEST}}\in B$.
$B_{\mathrm{HANDOVER}}$	The subset of APs involved in handovers. $B_{\mathrm{HANDOVER}}\subset B$.
$Q^{s}$	The quantum value of slice *s*.
$Q_{\mathrm{NEW}}^{s}$	The new quantum value calculated for slice *s*.
$Q_{\mathrm{MIN}}$, $Q_{\mathrm{MAX}}$	The minimum and maximum quantum value for slices.
$Q_{\mathrm{INC}}$, $Q_{\mathrm{DEC}}$	The increase and decrease factor for adapting The quantum value of slices.
$Q_{\mathrm{FACTOR}}$	The used factor for adapting The quantum value of a slice.
$\lambda^{t}$	The traffic shaping value for STA *t*.
$\lambda_{\mathrm{NEW}}^{t}$	The new traffic shaping value calculated for STA *t*.
$\lambda_{\mathrm{LOSS}}^{t}$	The loss introduced by The traffic shaping at STA *t*.
$\lambda_{\mathrm{MIN}}$, $\lambda_{\mathrm{MAX}}$	The minimum and maximum traffic shaping value of STAs.
$\lambda_{\mathrm{INC}}$, $\lambda_{\mathrm{DEC}}$	The increase and decrease factor for performing traffic shaping on STAs.
$\lambda_{\mathrm{FACTOR}}$	The used factor for performing traffic shaping of an STA.

**Table 4:** Multi-Criteria Decision Analysis (MCDA) criteria, objectives, and weights for AP *b*.

Criterion	Objective	$W_{\mathrm{BE}}$	$W_{\mathrm{QoS}}$	Description
$\theta^{b}$	MIN	0.05	0.10	Overall channel load of *b*.
$\mu^{b}$	MIN	0.10	0.10	Measured throughput of *b*.
$\mu_{\mathrm{EXP}}^{b}$	MIN	0.40	0.10	Overall expected throughput of *b*.
$D^{b}$	MIN	0.10	0.10	Measured average queueing delay of *b*.
$t_{\mathrm{RSSI}}^{b}$	MAX	0.15	0.20	Measured RSSI from STA *t* of *b*.
$t^{b}$	MAX	0.20	0.40	True if STA *t* is associated with *b*.

**Table 5:** Workload parameters used in experiment 1.

Scenario	Flow	STA	Direction	$\mu_{\mathrm{EXP}}^{s,t}$	$\mu_{\mathrm{QoS}}^{s}$/$\sum_{f\in F^{t}}\mu_{\mathrm{QoS}}^{f,t}$	$D_{\mathrm{QoS}}^{s}$
A	BE	1	UL	30 Mbps	N/A	N/A
	QoS	2	UL	15 Mbps	10 Mbps	N/A
B	BE	1	UL	30 Mbps	N/A	N/A
	QoS	2	DL	20 Mbps	N/A	30 ms
C	BE	1	DL	30 Mbps	N/A	N/A
	QoS	2	UL	15 Mbps	10 Mbps	N/A
D	BE	1	DL	30 Mbps	N/A	N/A
	QoS	2	DL	15 Mbps	N/A	30 ms

**Table 6:** Workload parameters used in experiment 2.

Event	Time (s)	Flow	STA	Direction	$\mu_{\mathrm{EXP}}^{s,t}$	$\mu_{\mathrm{QoS}}^{s}$/$\sum_{f\in F^{t}}\mu_{\mathrm{QoS}}^{f,t}$	$D_{\mathrm{QoS}}^{s}$
1	10	BE 3	3	DL	20 Mbps	N/A	N/A
		BE 4	4	DL	20 Mbps	N/A	N/A
2	70	BE 1	2	DL	20 Mbps	N/A	N/A
		BE 2	5	DL	20 Mbps	N/A	N/A
3	130	BE 3	3	DL	0 Mbps	N/A	N/A
		BE 4	4	DL	0 Mbps	N/A	N/A
4	190	QoS 1	1	DL	10 Mbps	10 Mbps	5 ms
		QoS 2	6	DL	8 Mbps	5 Mbps	100 ms
		BE 3	3	DL	30 Mbps	N/A	N/A
		BE 4	4	DL	30 Mbps	N/A	N/A

**Table 7:** Workload parameters used in experiment 3.

Event	Time (s)	Flow	STA	Direction	$\mu_{\mathrm{EXP}}^{s,t}$	$\mu_{\mathrm{QoS}}^{s}$/$\sum_{f\in F^{t}}\mu_{\mathrm{QoS}}^{f,t}$	$D_{\mathrm{QoS}}^{s}$
1	10	BE 3	5	DL	20 Mbps	N/A	N/A
		BE 4	6	DL	20 Mbps	N/A	N/A
2	70	BE 1	1	UL	20 Mbps	N/A	N/A
		BE 2	3	UL	20 Mbps	N/A	N/A
3	130	BE 3	5	DL	0 Mbps	N/A	N/A
		BE 4	6	DL	0 Mbps	N/A	N/A
4	190	QoS 1	4	DL	10 Mbps	10 Mbps	5 ms
		QoS 2	2	UL	10 Mbps	5 Mbps	N/A
		BE 3	5	DL	30 Mbps	N/A	N/A
		BE 4	6	DL	30 Mbps	N/A	N/A

## Data Availability

The data presented in this study are openly available in FigShare at https://doi.org/10.6084/m9.figshare.13607276, reference number 13607276.

## Abbreviations

The following abbreviations are used in this manuscript:

A-MSDU	Aggregated MAC Service Data Unit
ADWRR	Airtime Deficit Weighted Round Robin
AHP	Analytic Hierarchy Process
AP	Access Point
API	Application Programming Interface
ARP	Address Resolution Protocol
BE	Best-Effort
CDF	Comulative Ditribution Function
CSA	Channel Switch Annoucement
CUPS	Control/User Plane Split
CW	Contention Window
DCF	Distributed Coordination Function
DL	Downlink
DSCP	Differentiated Services Code Point
E2E	End-to-End
EDCA	Enhanced Distributed Channel Access
HD	High-Definition
HT	High Throughput
LVAP	Light Virtual Access Point
MAC	Medium Access Control
MCA	Mission-Critical Application
MCDA	Multi-Criteria Decision Analysis
MCS	Modulation and Coding Scheme
NIC	Network Interface Card
QoS	Quality of Service
QoSS	Quality of Service Slicing
RA	Resource Allocation
RAN	Radio Access Network
RAT	Radio Access Technology
RSSI	Received Signal Strength Indicator
SD-RAN	Software-Defined Radio Access Network
SDN	Software-Defined Networking
SLA	Service Level Agreement
SMA	Simple Moving Average
SMM	Simple Moving Median
SSID	Service Set Identifier
STA	Station
TCP	Transmission Control Protocol
TOPSIS	Technique for Order of Preference by Similarity to Ideal Solution
TXOP	Transmission Opportunity
UDP	User Datagram Protocol
UL	Uplink
URLLC	Untral-Reliably Low Latency Communication
VHT	Very High Throughput
